# Researches on the Medical Properties and Applications of Nitrous Oxide, Protoxide of Nitrogen, or Laughing Gas

**Published:** 1865-10

**Authors:** 


### Books Reviewed.

RESEARCHES ON THE MEDICAL PROPERTIES AND APPLICATIONS OF NITROUS OXIDE, PROTOXIDE OF NITROGEN, OR LAUGHING GAS. By GEORGE J. ZIEGLER, M. D., Physician to the Philadelphia Hospital, Member of the American Medical Association, Member of the Academy of Natural Sciences of Philadelphia, etc., etc. Revised and republished from the Medical and Surgical Reporter. Philadelphia: J. B. LIPPINCOTT & Co. 1865.

The author treats this substance under several heads: First, the "chemical constitution, properties and correlations of protoxide of nitrogen; second, its physiological influences and hygienic uses; third, its medical properties and applications, therapeutic, revivifying, antidotal and anæsthetic; fourth, its preparation and combinations; and fifth, its modes of administration and dose."

The enthusiasm of the author is unbounded, and he speaks of his hoppy-horse as though it should have been supplied instead of common air, being better suited to the wants of the animal economy than any other discovered substance. We will allow our author to speak for himself, and our readers to judge:

"In brief then, through its constituent elements and dynamic properties, nitrous oxide exerts a powerful influence in both supplying essential matter for organization, and in promoting the general molecular, cell, nutrient, reproductive and dynamic operations of the animal economy, those of the vegetal, animal, and psychical life inclusive. It is thus indeed, remarkably active and potent in promoting the various functions of digestion, absorption, circulation—both general and capillary—aeration or arterialization, hæmatosis, calorification, assimilation, disintegration, depuration, secretion, excretion, muscular and general contractility, innervation and intellection; and likewise, those of the reproductive system. Hence, for the preservation of the healthy integrity of the body and the regulation as well as invigoration of all the important functions of life, this agent may, *cæteris paribus*, be always employed with advantage. This concise but comprehensible outline of the sanitive effects and applications of protoxide of nitro gen will suffice to show that in an hygienic point of view it is both unique and invaluable. * * * * * * *

"Thus in comprehensive terms, nitrous oxide is a direct, potent and permanent chemico-organic, arterial, nervous, cerebral and general stimulant, secernent. depurant, aphrodisiac and antitoxic, having a special tendency to the blood, brain, nervous system and genito-urinary organs. It exerts a powerful invigorating influence over the entire economy and is a superior nutrient, hæmatic, neurotic, tonic, disintegrant, diuretic, disinfectant, alterative, resolvent, sorbefacient, antidote, antiseptic, etc., etc. Its primary action is usually prompt and frequently well marked, though somewhat transitory in character, while its secondary or more remote effects are permanent and highly salutary, the difference between them being more in degree than in kind, for, as before stated, the invigoration is generally continuous and persistent without subsequent depression, as is the case with most other stimulants. The properties and influences of nitrous oxide are, in other words, both organic and dynamic; organic in supplying the material elements—nitrogen and oxygen—for the various chemical purposes of the economy; and, dynamic in stimulating the functions of the whole body. Thus both through its constituent elements and dynamic influences it promotes the chemico-organic and dynamic operations of the animal organism, and while thereby regulating normal, resolves as well as prevents abnormal action. * * * * * * * * * * *

"In general nitrous oxide is of great utility in the treatment of those asthenic conditions in which the material and dynamical processes of the animal economy are in abeyance, and which are so frequently exhibited in the inertia of the various functions of the organism, those of the vegetal, animal and psychical life inclusive. It is therefore especially indicated in indigestion and inefficient absorption, as also in general inactivity of the chylopoietic functions; in imperfect aeration or arterialization of blood and deficient hæmatosis; in mal-assimilation and disintegration; in insufficient secretion and depuration; and, in irregular or defective mobility, contractility, innervation and cerebration. Hence in the various forms of asthenic dyspepsia and other morbid states dependent upon or associated with torpidity of the chylopoetic viscera; anæration, anæmatosis, and mal-nutrition generally, both primary and secondary; in depraved and defective secretion and depuration; in enervation, neuralgia, chorea, paralysis, melancholy, amentia and adynamic states generally, the nitrous oxide will, doubtless, always prove more or less useful as a curative agent.

"Protoxide of nitrogen is, moreover, strongly indicated in atonic conditions of the genito-urinary apparatus, more especially in inertia and such other abnormities of the urinary and reproductive organs as are presented in cases of incontinence and suppression of urine, paralysis of bladder, spermatorrhœa, impotence, sterility, some forms of amenorrhœa, dysmenorrhœa, leucorrhœa, menorrhagia, etc., as it has a special tendency to these organs, and exerts a powerful influence over their functions."

Perhaps it will not be worth while to continue our quotations. Sufficient has already been presented to show our readers the value of *protoxide of nitrogen*, according to the views of our author. Whoever desires to know more can learn all about it by reading this book.

